# A web-based scoping review assessing the influence of smoking and smoking cessation on antidiabetic drug meabolism: implications for medication efficacy

**DOI:** 10.3389/fphar.2024.1406860

**Published:** 2024-06-18

**Authors:** Carlo Maria Bellanca, Egle Augello, Giulia Di Benedetto, Chiara Burgaletto, Anna Flavia Cantone, Giuseppina Cantarella, Renato Bernardini, Riccardo Polosa, the DiaSmokeFree Working Group, Agostino Di Ciaula

**Affiliations:** ^1^ Department of Biomedical and Biotechnological Sciences, Section of Pharmacology, University of Catania, Catania, Italy; ^2^ Clinical Toxicology Unit, University Hospital of Catania, Catania, Italy; ^3^ Department of Clinical and Experimental Medicine, University of Catania, Catania, Italy; ^4^ Centre of Excellence for the Acceleration of HArm Reduction (CoEHAR), University of Catania, Catania, Italy; ^5^ Centre for the Prevention and Treatment of Tobacco Addiction (CPCT), University Hospital of Catania, Catania, Italy

**Keywords:** adverse drug reactions, cytochrome P450, diabetes mellitus, drug-drug interactions, drug metabolism, smoking cessation

## Abstract

Currently 1.3 billion individuals globally engage in smoking, leading to significant morbidity and mortality, particularly among diabetic patients. There is urgent need for a better understanding of how smoking influences antidiabetic treatment efficacy. The review underscores the role of cigarette smoke, particularly polycyclic aromatic hydrocarbons (PAHs), in modulating the metabolic pathways of antidiabetic drugs, primarily through the induction of cytochrome P450 (CYP450) enzymes and uridine diphosphate (UDP)-glucuronosyltransferases (UGTs), thus impacting drug pharmacokinetics and therapeutic outcomes. Furthermore, the review addresses the relatively uncharted territory of how smoking cessation influences diabetes treatment, noting that cessation can lead to significant changes in drug metabolism, necessitating dosage adjustments. Special attention is given to the interaction between smoking cessation aids and antidiabetic medications, a critical area for patient safety and effective diabetes management. This scoping review aims to provide healthcare professionals with the knowledge to better support diabetic patients who smoke or are attempting to quit, ensuring tailored and effective treatment strategies. It also identifies gaps in current research, advocating for more studies to fill these voids, thereby enhancing patient care and treatment outcomes for this at-risk population.

## 1 Introduction

Currently, 1.3 billion people globally use tobacco, predominantly through smoking, and this leads to over 7 million deaths annually due to smoking related illnesses (GBD, 2015 Tobacco Collaborators, 2017; Tobacco, 2024). Not only is smoking a primary factor in the development of lung cancer, chronic obstructive pulmonary disease (COPD), and cardiovascular diseases (Findings from the Global Burden of Disease Study, 2017 2024; GBD Compare, 2024), but it also contributes significantly to the onset of diabetes (Campagna et al., 2019). Furthermore, in individuals with diabetes, smoking exacerbates the detrimental effects of high blood glucose levels, thus hastening the progression of vascular damage (Knapp et al., 2019; Kondo et al., 2019).

The Global Burden of Disease (GBD) Study has estimated a significant reduction in the age-standardized prevalence of smoking globally among people in the general population from 1990 to 2019, with varying progress across countries and regions (GBD, 2019 Tobacco Collaborators, 2021). In contrast, the prevalence of smoking among individuals with diabetes has not seen a comparable decline over the same 30-year period (Noubiap et al., 2019; Roderick et al., 2019).

Abstinence from smoking can potentially delay the onset and slow the progression of diabetes-related complications, ultimately improving quality of life (Campagna et al., 2019). While the impact of smoking cessation on glycemic control, insulin resistance, and lipid parameters remains unclear (Walicka et al., 2022), smoking cessation has been shown to have beneficial effects in reducing the risks of cardiovascular disease, stroke, peripheral artery disease, and diabetic nephropathy in individuals with diabetes (Campagna et al., 2019). Therefore, prioritizing smoking cessation is vital for those with diabetes. The latest American Diabetes Association (ADA) guidelines underscore the significance of smoking cessation in the management of diabetes (ElSayed et al., 2023).

When addressing smoking cessation in individuals with diabetes, it is crucial to acknowledge that smoking—specifically, numerous chemicals in tobacco smoke—can modulate several liver enzymatic detoxification systems. This modulation may lead to substantial alterations in the pharmacokinetics of various antidiabetic drugs. Conversely, quitting smoking leads to a gradual normalization of these enzymatic detoxification systems’ function, significantly impacting drug pharmacokinetics. Consequently, in the course of smoking cessation, adjusting, or lowering the dosages of certain medications may be essential to ensure both effective and safe management.

**FIGURE 1:**
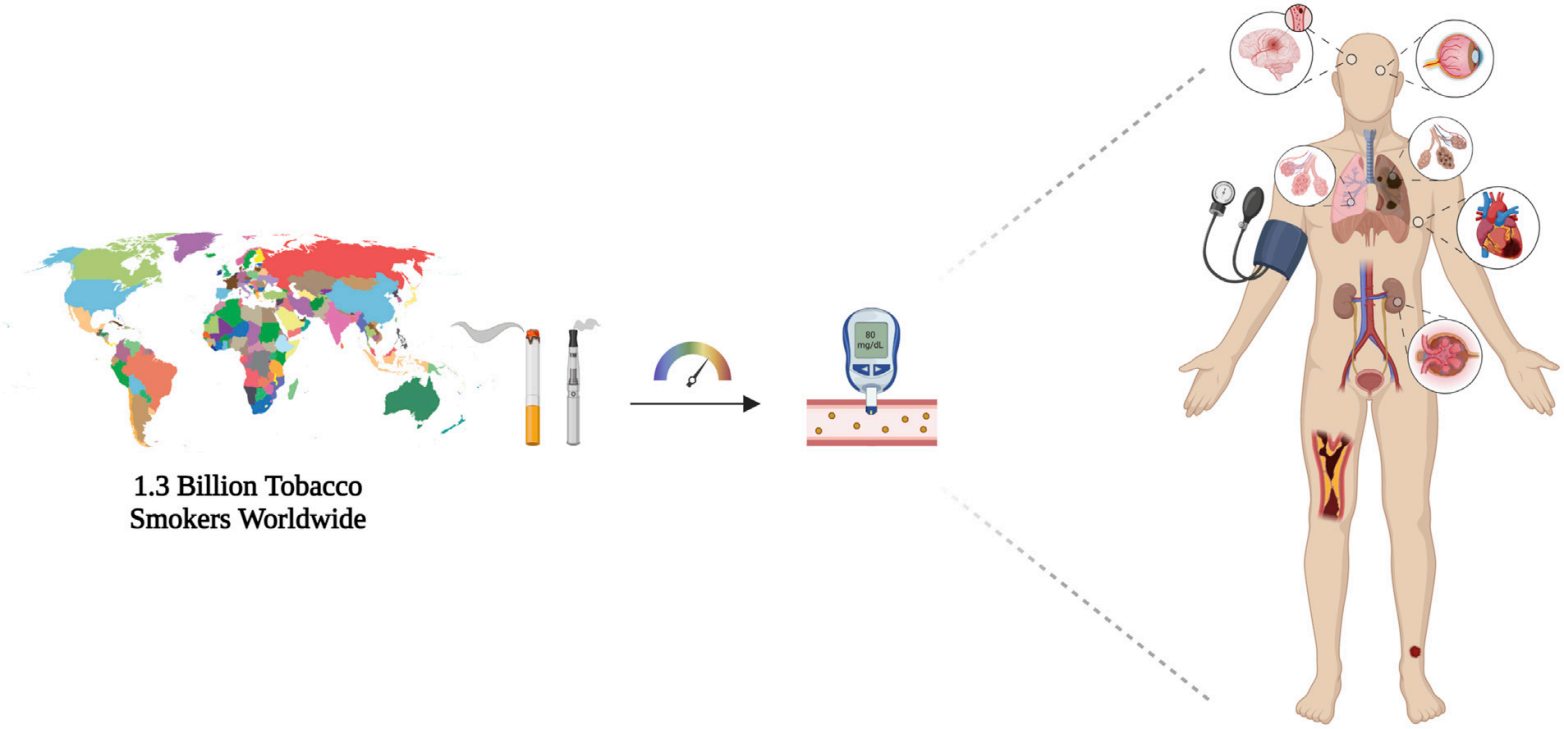
Schematic representation of number of tobacco smokers worldwide, which highlights the effect of smoking habit in accelerating type 2 diabetes-related consequences. Smoking worsens disease course altering glycometabolic parameters and long-term complications, namely diabetic retinopathy, cardiovascular diseases, and diabetic nephropathy. Created with BioRender.com; accessed on 12 March 2024.

This review article aims to examine the interactions between smoking, smoking cessation, and antidiabetic medications. It analyses how smoking habit influences the efficacy and pharmacokinetics of antidiabetic drugs. Furthermore, the article will also discuss the potential interactions between medications used in smoking cessation programs and antidiabetic drugs. By shedding light on these dynamics, the review aims to provide critical insights for healthcare professionals in optimizing treatment strategies for patients with diabetes mellitus who are current or former smokers and to identify new areas for future research (Figure 1).

## 2 Materials and methods

Studies (i.e., observational studies, randomized and non-randomized clinical trials, experimental studies, case report, and case series) that contained useful information about the potential metabolic interactions of smoking, smoking cessation, and smoking cessation medications on pharmacokinetics and pharmacodynamics of antidiabetic drugs were included.

An extensive review of the literature was carried out from October 2023 to January 2024. The search was conducted across MEDLINE (PubMed) and EMBASE. Search queries were formulated by experts from the DiaSmokeFree Working Group. Tailored search strings were created and utilized to find pertinent studies. Search strings details are reported in Supplementary Material. Others specific databases such as DrugBank.com (DrugBank, 2024b) and PharmGKB (2024) as well as Summary of Product Characteristics (SmPC) were also consulted for each medication.

CMB, EA, and GDB screened each title and abstract to select studies for full-text review. Studies that either seemed appropriate for inclusion or those that could not clearly be excluded on title and abstract alone were carried forward for further evaluation. The software Zotero (Zotero 6.0.30; Roy Rosenzweig Centre for History and New Media (RRCHNM); George Mason University) was used to manage records and duplicates. Then, three authors (CB, AFC, and GC) independently assessed each remaining full-text article to determine eligibility. Disagreement was resolved by discussion and consensus with Experts from DiaSmokeFree Working Group. Articles meeting the specified inclusion criteria were chosen for inclusion in the qualitative synthesis. Moreover, the references of the included articles and review papers underwent additional screening to identify any potentially relevant articles.

Extraction of relevant data was performed by using a standardized data extraction form designed explicitly for this purpose. The extracted information, including author, study population, study design, definition of smoking status, median duration of follow up, and results, was systematically recorded and organized in a tabular format. In the qualitative synthesis, emphasis was placed on the clinical impact of smoking abstinence on diabetes-related complications.

## 3 Impact of tobacco smoke on enzymatic drug metabolism

Cigarette smoke is a complex mixture of chemicals, including toxic and carcinogenic substances such as polycyclic aromatic hydrocarbons (PAHs), ammonia, aromatic amines, phenols, carbonyls, hydrocyanic acid, and N-nitrosamines (Hoffmann, 1997, 1950–95). From a chemical standpoint, tobacco smoke is composed of two distinct phases, gaseous and particulate. The particulate phase harbours the majority of >7,000 chemicals identified in tobacco smoke, including nicotine, PAHs, tars, pigments, and nitrosamines (Dawson and Vestal, 1981). Volatile constituents like carbon monoxide (CO), hydrogen cyanide, and aldehydes are present at the gaseous phase (Borgerding and Klus, 2005). Several of these substances interact with the biosynthetic functions of enzymes responsible for xenobiotic metabolism and various transporters, thereby affecting the biotransformation of drugs. This suggests that cigarette smoking, as well as cessation, can impact drug metabolism (Maideen, 2019).

Nicotine and PAHs are among the most well-documented compounds found in tobacco products. More than five hundred different PAHs have been identified in tobacco smoke—compounds consisting of two or more fused benzenoid rings, reported as carcinogenic and mutagenic agents (McAdam et al., 2013). They do not occur naturally in tobacco plants, but are formed mainly by the incomplete combustion of tobacco and other organic constituents during smoking (Stepanov et al., 2010).

PAHs primarily influence metabolism by inducing the activity of hepatic cytochrome P450 (CYP450) enzymes, a family of haemoproteins involved in phase I (oxidative) reactions of drug biotransformation and xenobiotic detoxification (McDonnell and Dang, 2013). Additionally, uridine diphosphate (UDP)-glucuronosyltransferases (UGTs), enzymes responsible for phase II (conjugative) reactions, particularly glucuronidation, can also be affected by PAHs (Meech and Mackenzie, 1997). Specific CYP450 isoenzymes, such as CYP1A1, 1A2, 1B1, and 2E1, may be implicated (Meech and Mackenzie, 1997). As PAHs increase the activity of CYPs, drugs metabolized by these enzymes are cleared more rapidly, resulting in decreased blood concentrations. This would require careful therapeutic monitoring and potential dose adjustment. Furthermore, a growing body of evidence suggests changes in enzyme activity, possibly through epigenetic mechanisms, with persistent accelerated metabolism even after smoking cessation (Hirota et al., 2008; O’Malley et al., 2014).

Clinically relevant is the induction of CYP1A2 by tobacco smoke, given its role in the metabolism of numerous drugs. A clinical study aimed at estimating CYP1A2 activity, assessed caffeine clearance in 863 healthy Caucasians, taking tobacco habit into account among the covariates. A 1.66-fold increase in CYP1A2 activity was observed for smokers consuming 11–20 cigarettes daily (Tantcheva-Poór et al., 1999). Consistent with caffeine clearance assessment, the induction of CYP1A2 by cigarette smoking is reversible after cessation. Indeed, within the first 4 days of quitting, initial caffeine clearance decreased significantly by 36% (Faber and Fuhr, 2004). Thus, for individuals with a history of heavy smoking and undergoing treatment with drugs metabolized by CYP1A2, particularly those with a narrow therapeutic index, a dosage adjustment post-cessation is critical to avoid increased systemic exposure and potential adverse drug reactions (ADRs).

CYP1A1, another enzyme influenced by smoking, is regulated by aryl hydrocarbons in cigarette smoke, like benzo [a]pyrene, benzo-fluorene, tetrachlorodibenzo-p-dioxin, and fluoranthene. Studies have shown an approximate 66%–70% increase in CYP1A1 activity among smokers (Vistisen et al., 1991). Similarly, tobacco consumption has been linked to increased CYP1B1 mRNA expression, with its induction potentially affected by genetic polymorphisms (Helmig et al., 2010).

Tobacco smoke also enhances CYP2E1 expression and activity (Seree et al., 1996; Villard et al., 1998), critical for metabolizing substances such as chlorzoxazone. A study by Benowitz et al. (Benowitz et al., 2003) demonstrated a 24% acceleration in chlorzoxazone metabolism in smokers, highlighting the enzyme’s induction through smoking. In contrast, the impact of UGTs presents mixed findings. While some studies report variable effects of smoking on UGT-mediated drug glucuronidation (Fleischmann et al., 1986), others have identified significant increases in UGT activity in smokers, particularly in placental tissue during pregnancy (Collier et al., 2002). Further studies showed higher activity of UGT1A4 and UGT1A6 in smokers compared to non-smokers, whereas UGT1A1 and UGT2B7 activities were unaffected (Dragacci et al., 1987; Court, 2010). This variability suggests that smoking may differentially affect UGT isoforms, warranting further investigation to clarify these effects.

Overall, the presence of PAHs in cigarette smoke significantly alters drug metabolism by modifying the activity of critical biotransformation enzymes, such as CYPs and possibly UGTs. These effects necessitate careful patient management, especially among smokers or those in the process of quitting, to ensure optimal pharmacotherapy. The reversibility of enzyme induction post-smoking cessation further emphasizes the need for vigilant monitoring and drug dosage adjustments in quitters.

Approximately 70%–80% of nicotine is metabolized into cotinine, mainly by the activity of CYP2A6 and CYP2B6 (Nakajima et al., 1996; Bao et al., 2005; Mwenifumbo and Tyndale, 2009). Tobacco smoke has been shown to inhibit CYP2A6, leading to a decrease or at least a slowdown in nicotine metabolism in smokers compared to non-smokers (Lee et al., 1987; Benowitz and Jacob, 1993; Benowitz and Jacob, 2000).

Nicotine not only serves as a substrate for metabolizing enzymes and drug transporters, but also appears to alter their activity. Several studies have indicated that nicotine may interfere with Organic Cation Transporter (OCT) proteins in transporting drugs to organs and tissues (Bergen et al., 2014). Nicotine has been shown to inhibit the accumulation of tetraethylammonium (a model substrate for OCTs) in a human embryonic kidney cell line (HEK-293), mediated by OCT1 with an IC50 of 63 μM and by OCT2 with an IC50 of 50 μM *in vitro* (Urakami et al., 1998). Furthermore, Lips et al. found an IC50 of 42 μM for nicotine with OCT2 expressed in *Xenopus* oocytes (Lips et al., 2005). However, the impact of nicotine on CYP450 isoforms remains contentious. *In vivo* experiments have demonstrated that nicotine can induce the activity of various enzymes within the central nervous system (CNS), including CYP2E1, CYP2A1/2A2, and CYP2B1/2B2 (Anandatheerthavarada et al., 1993a; Anandatheerthavarada et al., 1993b), but the clinical relevance of these findings is still uncertain (Zevin and Benowitz, 1999).

In 2009, Hukkanen et al. performed a single-blind, randomized, crossover two-arm study to assess the effects of nicotine on the disposition kinetics of intravenously infused deuterium-labelled nicotine and cotinine and oral chlorzoxazone, verifying the hypotheses that nicotine reduces its own CYP2A6-mediated metabolism and induces CYP2E1 activity. Results showed that nicotine is not responsible for the effects of tobacco smoke on these isoenzymes (Hukkanen et al., 2010). Although interactions between pharmacokinetic drugs and tobacco smoke are primarily attributed to PAHs rather than nicotine, the latter is significantly involved in pharmacodynamic interactions through the activation of the sympathetic nervous system (Benowitz, 1997). This mechanism may negate the pharmacological effects of some medications.

Beyond PAHs and nicotine, other substances such as acetone, pyridine, heavy metals, benzene, and CO in tobacco smoke may also interact with hepatic enzymes, albeit their impact is considered less significant (Kroon, 2007).

## 4 Effects of tobacco smoke on metabolism of antidiabetic medications

The treatment options for type 2 diabetes mellitus (T2DM) include various drug classes such as sulfonylureas (SUs), glinides (or meglitinides), biguanides, thiazolidinediones (also known as glitazones), α-glucosidase inhibitors, dipeptidyl peptidase-4 (DPP-4) inhibitors, glucagon-like peptide-1 (GLP-1) receptor agonists, and sodium-glucose linked transporter-2 (SGLT-2) inhibitors.

The pharmacokinetics and pharmacodynamics characteristics of such antidiabetics may differently affect the potential for the occurrence of drug-drug interactions (DDIs) with tobacco smoking compounds.

SUs are mainly metabolised hepatically, in particular by CYP2C9 and to a lesser extent by CYP3A4, whereas gliclazide is substrate for CYP2C19. They also may be substrates for drug transporters, in particular P-glycoprotein. Glyburide and glimepiride are by far the most frequently prescribed SUs (Skillman and Feldman, 1981), displaying a half-life of between 5 and 10 h, but their pharmacological effect may exceed because of the active compounds derived from hepatic metabolism (Holstein and Beil, 2009).

The glinides include the compounds repaglinide (Moses et al., 1999) and mitiglinide (Sunaga et al., 2001). After oral administration, repaglinide is rapidly absorbed (van Heiningen et al., 1999) and has a bioavailability of approximately 63% (Hatorp et al., 1998). It is metabolised in the liver to inactive metabolites and is predominantly excreted via the bile into the faeces, while only a small proportion of the parent compound appears in the urine (Kajosaari et al., 2005). Repaglinide is partly metabolised through CYP3A4, CYP2C8, and CYP2C9. The principal transporter of glinides is the Organic Anion Transporting Polypeptide (OATP) 1B1. Only minor pharmacokinetic drug–drug interactions have been observed with meglitinides. Reported increases in Area Under the Curve (AUC) with co-administration of drugs inhibiting CYP450 isoenzymes never exceeded 80% for repaglinide (Scheen, 2007).

Metformin represents the first-line treatment for T2DM since 1957, from the point of diagnosis onwards (Nathan et al., 2009; Zachou et al., 2024). Half-life is reported to be between 1.5 and 5 h. It does not tend to accumulate in the liver as a result of its modest lipophilicity. The metformin binding to plasma proteins is negligible and it is eliminated unmodified by active tubular secretion and glomerular filtration (Scheen, 1996), thus is contraindicated in patients with kidney failure (Contraindications can damage your health--is metformin a case in point?, 2024). CYP450 is not involved in its metabolism, indeed, oxidative or conjugated metabolites of metformin have not been identified in any biological samples, including plasma, urine, or feces. Nevertheless, the potential for DDIs is rooted in the fact that metformin is a substrate of at least two OATs encoded by the SLC22A gene family (Kimura et al., 2005). It is highly recommended that patients taking cationic medications that are excreted through the proximal renal tubular secretory system undergo careful monitoring and dose adjustment of metformin and/or the interfering drug (Glucophage/Glucophage Forte/Risidon/Dianben - referral | European Medicines Agency, 2024).

Although the majority of cases of lactic acidosis occur in elderly patients with multiple comorbidities, cases have been documented in the absence of renal impairment. Therefore, it would be appropriate to review the continued use of metformin in patients with an estimated glomerular filtration rate (eGFR) < 45 mL/min and to discontinue the drug if the eGFR is <30 mL/min (Bruijstens et al., 2008; National Collaborating Centre for Chronic Conditions UK, 2008).

Acarbose is by far the most widely prescribed α-glucosidase inhibitor. Acarbose is poorly absorbed from the gastrointestinal tract, with less than 2% of the administered dose being absorbed in unchanged form. Its bioavailability is low because it exerts its pharmacological activity locally in the gastrointestinal tract, where acarbose is predominantly metabolized. The biotransformation process involves the intestinal microflora and, at a lower level, digestive enzymes. By means of chromatography, at least 13 metabolites have been isolated from urine samples. DDIs are quite unlikely, but cannot be excluded (Holstein and Beil, 2009).

Thiazolidinediones, also known as glitazones, are a class of oral antidiabetic agents that were initially developed in the early 1980s as antioxidants (Yoshioka et al., 1989). Pioglitazone is the major component of this class, also commercialized in combination with others oral antidiabetics (Research, 2022). Oral bioavailability of this compound has been shown to be approximately 83%, unaffected by the presence of food in the gastrointestinal tract (Karim et al., 2007). Significant risk of fluid and salt retention make it contraindicated in patients with heart failure, doubling the risk of decompensation regardless of age and dosage (Singh et al., 2007).

Up to date, three SGLT-2 inhibitors are used in Europe, namely canagliflozin, dapagliflozin, and empagliflozin (Zachou et al., 2024).

Currently, a number of GLP-1 receptor agonists have been approved for the treatment of T2DM, namely exenatide, liraglutide, dulaglutide, and semaglutide. GLP-1-based therapies with a longer half-life than that of the native compound were developed in order to exploit the enhancing of insulin secretion, the decreasing of glucagon secretion, as well as the regulation of satiety and appetite and gastrointestinal motility (Meier, 2012; Holst, 2007, 1). Indeed, modifications in the molecule structure of GLP-1 receptor agonists made them resistant to enzymatic degradation exerted by DPP-4. GLP-1 receptor agonists that have been approved for marketing are derivatives of either human incretine hormone GLP-1 or exendin-4 (a lizard peptide with 53% homology to human GLP-1) (Cheang and Moyle, 2018). Based on pharmacokinetic properties, these medications can be categorised into short- and long-acting. The former have a half-life of 2–4 h, whereas the latter have a half-life greater than 12 h, allowing for once-weekly administration (Uccellatore et al., 2015; Tomlinson et al., 2016; Anderson, 2018). Besides semaglutide, which has the potential to be orally administered, all GLP-1 receptor agonists are available in subcutaneous formulations. GLP-1 receptor agonists do not engage in CYP450 or transporter-mediated DDIs (Neumiller, 2011). However, it remains unclear whether their effect on gastric emptying might modulate the rate of drug absorption. This might be of clinical importance, mainly for drugs with a narrow therapeutic index, as small differences in blood concentration may lead to treatment failure or ADRs (Calvarysky et al., 2024). The majority of pharmacokinetic studies have so far shown that the absorption profiles of oral drugs are unaffected, making it unsurprising that co-administration of a GLP-1 receptor agonist did not influence AUC (Malm-Erjefält et al., 2015; Tham et al., 2018). Furthermore, even when the AUC was reduced no clinically significant effect was observed (Pinelli et al., 2013; Hausner et al., 2017). However, such studies are subject to a significant limitation, the recruitment of healthy individuals. Bearing in mind that the target population benefiting from GLP-1 receptor agonists are diabetics and obese patients, who often suffer from co-morbidities that may alter the results.

Up till now, several DPP-4 inhibitors have been authorized by regulatory agencies, namely sitagliptin, vildagliptin, saxagliptin, and linagliptin. Sitagliptin is rapidly absorbed and exhibits a half-life of 8–14 h. CYP3A4 is the major enzyme implicated in the marginal hepatic metabolism of sitagliptin (Januvia, 2024), as about 79% is excreted through active secretion in urine (Vincent et al., 2007). *In vitro* studies did not find inhibitory activity on CYP450 enzymes, but human studies are lacking (Mistry et al., 2008). However, preclinical data reveal that sitagliptin is carried by the renal absorption transporter hOAT3 and also serves as substrate for OATP4C1 and P-glycoprotein (Chu et al., 2007).

A critical, but often overlooked, issue is the interaction between antidiabetic medications and smoking cessation. This interaction can complicate diabetes management and interfere with the success of quitting smoking. The effects of smoking on the metabolism of antidiabetic drugs, primarily through the induction and, to a lesser extent, inhibition of CYP450 enzymes, are central to this issue. Understanding these effects is crucial, given the significant number of people affected by T2DM who also smoke. Smoking cessation can alter these metabolic processes, increasing the risk of ADRs or lack of therapeutic efficacy (Kroon, 2007).

While the interactions between tobacco smoking and medications such as antipsychotics, antidepressants, and anticoagulants are relatively well-documented, especially in certain groups of individuals (Bellanca et al., 2023), the research on interactions between smoking and antidiabetic drugs is limited. Among the oral antidiabetic medications currently available, pioglitazone, dapagliflozin, and canagliflozin are known, like other drugs, to be metabolized by CYP450 enzymes, which can be affected by compounds found in cigarette smoke.

Pioglitazone undergoes extensive metabolism in the liver through hydroxylation and oxidation, leading to four primary metabolites (M-I, M-II, M-IV, and M-V) and two secondary metabolites (M-III and M-VI), with M-III being derived from M-IV (Eckland and Danhof, 2000; Lin et al., 2003). The involvement of human CYP450 enzymes in pioglitazone metabolism has been somewhat debated. *In vitro* studies indicate that CYP2C8 primarily metabolizes pioglitazone, with a lesser role for CYP3A4 (Jaakkola et al., 2006). Moreover, latest findings still suggest CYP3A4’s role is minor, highlighting CYP2C8, CYP1A2, and CYP2D6 as key enzymes in converting pioglitazone to M-IV (Muschler et al., 2009). Given the effect of PAHs on enhancing CYP1A2 activity, diabetic smokers may experience increased metabolite synthesis, potentially necessitating dosage adjustments.

Dapagliflozin mainly undergoes glucuronidation by UGT enzymes, particularly UGT1A9 (Kasichayanula et al., 2014). The metabolism involves various CYP450 enzymes and UGTs, with UGT1A9 playing a crucial role in glucuronidation (Meng et al., 2008). The effects of PAHs on CYP1A1, UGT1A9, and the inhibition of CYP2A6 highlight the need for dose adjustments and monitoring in diabetic smokers.

**FIGURE 2:**
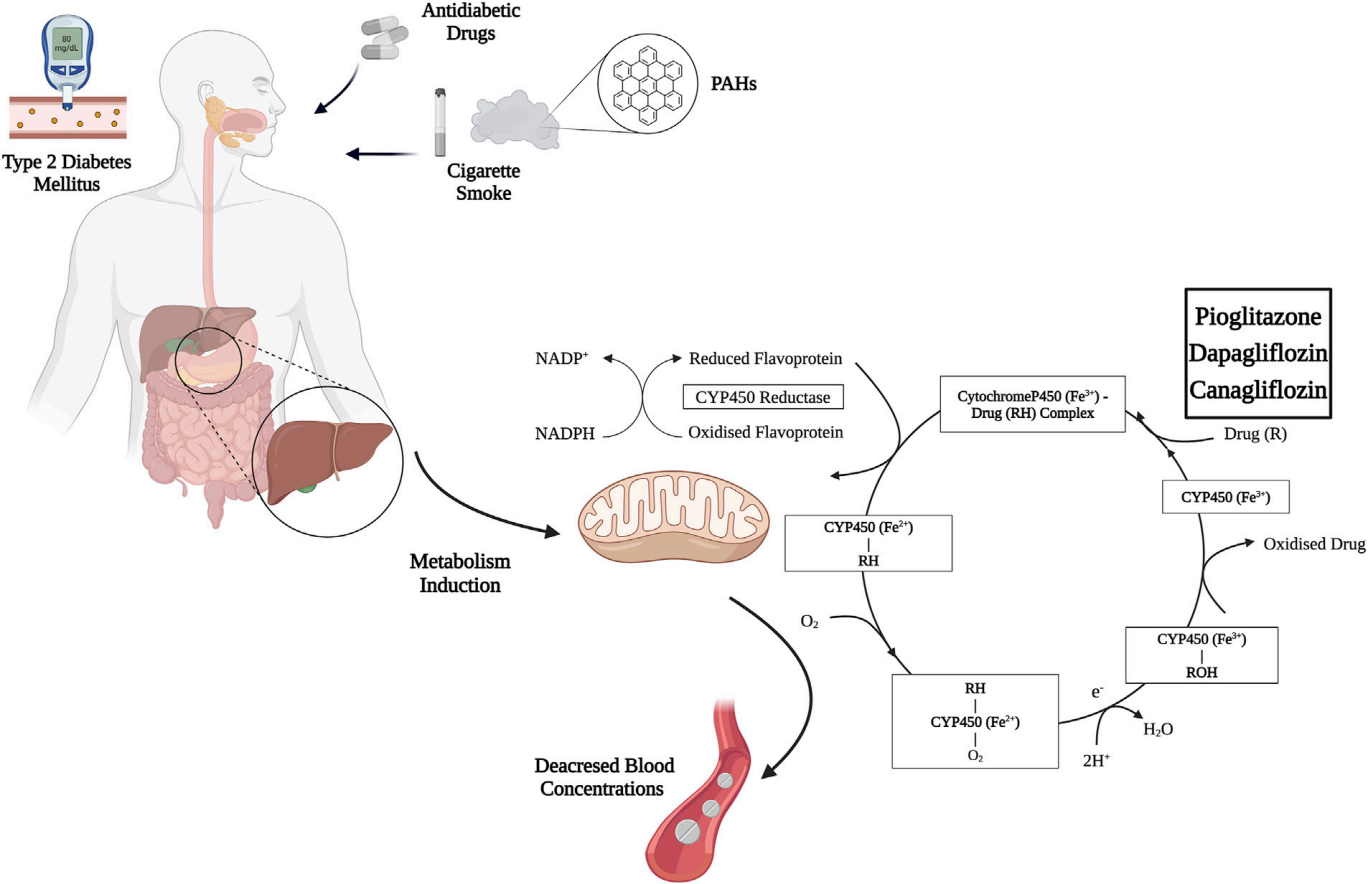
Mechanism of induction of pioglitazone, canagliflozin, and dapagliflozin metabolism by cigarette smoke PAHs. Abbreviations: PAHs, polycyclic aromatic hydrocarbons; NADP+, nicotinamide adenine dinucleotide phosphate; NADPH, Nicotinamide Adenine Dinucleotide Phosphate Hydrogen. Created with BioRender.com; accessed on 15 March 2024.

Canagliflozin is primarily glucuronidated by UGT1A9 and UGT2B4, forming inactive O-glucuronide metabolites, with minimal oxidative metabolism by CYP3A4 (Invokana, 2024). The induction of UGT1A9 by PAHs suggests that monitoring glycometabolic parameters in patients may be advisable. Table 1 and Figure 2 provide an overview of the main cytochromes involved in the metabolism of pioglitazone, canagliflozin, and dapagliflozin and possible interactions with tobacco smoke.

**TABLE 1:** Cytochromes involved in the metabolism of antidiabetic drugs and possible effects of tobacco smoke compounds on their metabolic activities.

Drug Class	Drug	CYP450 Metabolic Pathway Involved (Ref.)	Cigarette Smoke Effects on CYP450 Activities (Ref.)
Sulfonylureas	Glimepiride	CYP2C9 Agenzia Italiana del Farmaco (2024)	Smoking does not affect the activity of CYP2C9 (Kim et al., 2006)
	Glipizide	CYP2C9 (Kidd et al., 1999)	Smoking does not affect the activity of CYP2C9 (Kim et al., 2006)
	Glyburide	CYP2C9 (also CYP3A4/5, CYP3A7, and CYP2C19) Agenzia Italiana del Farmaco (2024); Ravindran et al., 2006; Shuster et al., 2014)	CYP3A1 can be inhibited significantly by long-term smoking in rats. Therefore, it indicates the possible inhibition of CYP3A4 activities in smokers (He et al., 2015). However, a previous *in vitro* study highlighted that PAHs activate CYP3A4 gene transcription through the activation of hPXR in HepG2 cells. Thus, PAHs may contribute to CYP3A4 induction in human liver (Kumagai et al., 2012). Smoking does not affect the activity of CYP2C9 (Kim et al., 2006)
	Gliclazide	CYP2C19 (Zhang et al., 2007)	No influence of smoking habit on CYP2C19 enzyme activity (Ramsjö et al., 2010)
Glinides	Mitiglinide	—	—
	Repaglinide	CYP3A4, CYP2C9, and CYP2C8 (Bidstrup et al., 2003; Anti-Diabetic Drug Repaglinide Pathway, Pharmacokinetics (2024); Repaglinide (2024)	CYP3A1 can be inhibited significantly by long-term smoking in rats. Therefore, it indicates the possible inhibition of CYP3A4 activities in smokers (Ramsjö et al., 2010). However, a previous *in vitro* study highlighted that PAHs activate CYP3A4 gene transcription through the activation of hPXR in HepG2 cells. Thus, PAHs may contribute to CYP3A4 induction in human liver (Kumagai et al., 2012). Smoking does not affect the activity of CYP2C9 (Kim et al., 2006)
Biguanides	Metformin	—	—
Thiazolidindions	Pioglitazone	CYP2C8, CYP3A4, CYP1A2, and CYP2D6 (Jaakkola et al., 2006; Muschler et al., 2009)	Carbon monoxide (CO) is an *in vitro* inhibitor of CYP enzymes. The effect of CO on the CYP system has been mainly studied in animal tissues and in perfused organs. There is a dose-effect response: the higher is CO concentration, more pronounced is the inhibition. The inhibition seems to be a direct effect of CO on the metabolizing enzymes, rather than nonspecific effects of tissue hypoxia. Moreover, the inhibition of CO to CYP enzymes is selective, indeed, in human liver microsomes CO inhibited CYP2D6, but not CYP2C or CYP3A activity. However, the concentrations of CO used in those studies were much higher than those to which the average smoker is exposed. Nonetheless, Montgomery and Rubin achieved significant inhibition of hexobarbital clearance in rats with CO concentrations comparable with those to which smokers are exposed (1,000–2,500 ppm) Zevin and Benowitz (1999)
α-glucosidase inhibitors	Acarbose	—	—
GLP-1 Receptor Agonist	Exenatide	—	—
	Liraglutide	—	—
	Dulaglutide	—	—
	Semaglutide	—	—
DPP-4 Inhibitors	Sitagliptin	CYP3A4 (to a lesser extent by CYP2C8) Agenzia Italiana del Farmaco (2024)	CYP3A1 can be inhibited significantly by long-term smoking in rats. Therefore, it indicates the possible inhibition of CYP3A4 activities in smokers (He et al., 2015). However, a previous *in vitro* study highlighted that PAHs activate CYP3A4 gene transcription through the activation of hPXR in HepG2 cells. Thus, PAHs may contribute to CYP3A4 induction in human liver (Kumagai et al., 2012)
	Linagliptin	CYP3A4 (Blech et al., 2010; DrugBank 2024b)	CYP3A1 can be inhibited significantly by long-term smoking in rats. Therefore, it indicates the possible inhibition of CYP3A4 activities in smokers (He et al., 2015). However, a previous *in vitro* study highlighted that PAHs activate CYP3A4 gene transcription through the activation of hPXR in HepG2 cells. Thus, PAHs may contribute to CYP3A4 induction in human liver (Kumagai et al., 2012)
	Vildagliptin	—	—
	Saxagliptin	CYP3A4/5 Saxagliptin (2024); Agenzia Italiana del Farmaco (2024)	CYP3A1 can be inhibited significantly by long-term smoking in rats. Therefore, it indicates the possible inhibition of CYP3A4 activities in smokers (He et al., 2015). However, a previous *in vitro* study highlighted that PAHs activate CYP3A4 gene transcription through the activation of hPXR in HepG2 cells. Thus, PAHs may contribute to CYP3A4 induction in human liver (Kumagai et al., 2012)
SGLT-2 Inhibitors	Canagliflozin	CYP3A4 (negligible) DrugBank (2024b); Agenzia Italiana del Farmaco (2024)	CYP3A1 can be inhibited significantly by long-term smoking in rats. Therefore, it indicates the possible inhibition of CYP3A4 activities in smokers (He et al., 2015). However, a previous *in vitro* study highlighted that PAHs activate CYP3A4 gene transcription through the activation of hPXR in HepG2 cells. Thus, PAHs may contribute to CYP3A4 induction in human liver (Kumagai et al., 2012)
	Empagliflozin	—	—
	Dapagliflozin	CYP1A1, CYP1A2, CYP2A6, CYP2C9, CYP2D6, CYP3A4 (CYP-mediated metabolic process represented a secondary clearance pathway in humans) (Kasichayanula et al., 2014, 2; Obermeier et al., 2010)	Polycyclic aromatic hydrocarbons (PAHs) induce CYP1A1 and CYP1A2 activity. Tobacco smoke appears to inhibit CYP2A6 Anderson and Chan (2016). Smoking does not affect the activity of CYP2C9 (Kim et al., 2006)

## 5 Interactions between smoking cessation medications and antidiabetic drugs

DDIs can significantly impact the efficacy and safety of treatment regimens of oral antidiabetic drugs in patients affected by T2DM with comorbidities (Utami and Octavia, 2022). Discussing the relationship between smoking and diabetes, it is important to consider any potential interactions between smoking cessation medications and antidiabetic drugs. Smoking cessation medications, when combined with behavioral support, can be effective in helping motivated smokers to quit (Stead et al., 2016; Giulietti et al., 2020). Although these drugs are generally well tolerated, this aspect in diabetic smokers is not well documented. Whether smoking cessation medications affect the metabolism and effectiveness of antidiabetics, it may necessitate adjustments in dosages of the latter to maintain appropriate therapeutic levels and ensure effective management of diabetes.

Approved first-line medications for smoking cessation include various forms of nicotine-replacement therapy (NRT), bupropion, and varenicline (Polosa and Benowitz, 2011). Cytisine is available for use in 18 countries but is not currently approved by the US Food and Drug Administration (FDA) or the European Medicines Agency (EMA). Varenicline, bupropion, and NRT have all been shown to improve quit rates in the general population of smokers (Cahill et al., 2013). Compared with placebo, the likelihood of quitting smoking was roughly doubled with NRT (1.84, 95% CI 1.71–1.99) and bupropion (1.82, 1.60–2.06) and was improved further with varenicline (2.88, 2.40–3.47). However, data on safety and efficacy of these pharmacotherapies in people with T2DM is limited (Nagrebetsky et al., 2014).

The management of DDIs largely depends on the clinical impact and severity of the interaction, many tools are available to determine their clinical significance. However, there is poor agreement among the current resources and a standardized classification method would be warranted. More specifically, the British National Formulary marks with bullet points potentially harmful drug pairs which should be prescribed cautiously, under appropriate monitoring, or avoided altogether (British National Formulary BNF, 2024). Micromedex Drug–Reax System categorizes interactions into three degrees of severity, major, moderate, and minor, and the strength of the reporting into five categories—excellent, good, fair, poor, and unlikely (DRUG-REAX, 2023). Drugs.com Drug Interaction Checker (DDIC) and DrugBank.com classify interactions into four severity levels: major, moderate, minor, and unknown (DrugBank, 2024b; Drug Interaction Checker, 2024). Vidal’s Interactions médicamenteuses comprises four seriousness grades according to the recommended clinical management—contraindicated, avoid, precaution, and “take into account” (i.e., no specific recommendation) (Les interactions médicamenteuses, 2023). Drug Interaction Facts rates interaction severity into three levels—major, moderate, and minor—and the degree of documentation into five—established, probable, suspected, possible, and unlikely—by combining these two categories. It also ranks each interaction from 1 to 5 in terms of importance (Tatro, 2014).

While dedicated research on the interactions between NRT and antidiabetic drugs are scarce, the capacity of nicotine—and by extension, NRT—to raise blood glucose levels, disrupt glucose homeostasis, and lead to insulin resistance could be taken into account (Chen et al., 2023). However, studies indicate that smokeless tobacco products, which deliver high levels of nicotine, do not increase the risk of diabetes (Natvig Norderhaug et al., 2005; Arabi, 2006).

As highlighted previously nicotine may interfere with OCT proteins in transporting drugs to organs and tissues (Bergen et al., 2014). Particularly, online resources, such as DrugBank.com (DrugBank, 2024b), medstopper.com (MedStopper, 2024), drugs.com (Drug Interaction Checker, 2024), and intercheckweb. marionegri.it (INTERCheck WEB, 2024), which are employed in clinical setting for the evaluation of DDIs, report that plasma concentration of metformin and linagliptin can be increased when it is combined with nicotine. More specifically DrugBank.com reports that the subject drug (nicotine) is an OCT2 inhibitor, and the affected drugs (metformin and linagliptin) are OCT2 substrates. Therefore, co-administration of these medications may increase plasma concentrations of OCT2 substrates, increasing the risk of adverse reactions.

There are limited studies specifically investigating the interactions between varenicline and antidiabetic medications, leaving this area underexplored. *In vitro* studies indicate that varenicline is unlikely to alter the pharmacokinetics of compounds that are primarily metabolized by CYPs. Moreover, as metabolism of varenicline accounts for less than 10% of its clearance, agents known to affect the CYP450 system are not expected to affect the pharmacokinetics of varenicline and therefore dose adjustment would not be required. Furthermore, at therapeutic concentrations, varenicline is not an inhibitor of human renal transport proteins, as preclinical studies have shown. Thus, active substances that are cleared by renal secretion (e.g., metformin) are unlikely to be modified by varenicline (EMA Champix, 2023). However, online resources, report that sitagliptin may decrease the excretion rate of varenicline. In turn, varenicline may reduce the clearance of saxagliptin. In both instances, renal excretion of drugs is the overall result of a combination of kidney processes, including glomerular filtration, passive diffusion, tubular secretion, and reabsorption. Since two of these mechanisms–tubular secretion and reabsorption–are saturable processes, they are susceptible to competition between multiple substrates for elimination. If two or more predominantly renally excreted drugs are co-administered, they may compete at this level; there is a high probability that one agent will “out-compete” or saturate the renal excretion mechanisms before the other co-administered agent(s) is/are eliminated. As a result, clearance of the concomitantly administered agent(s) may be inhibited or otherwise delayed, which could lead to an increase in their serum concentrations, increasing the risk and/or severity of adverse reactions associated with exposure to such drugs (DrugBank, 2024b). Notably, information from digital tools must always be compared with SmPC of interacting drugs in order to avoid errors or misinterpretations. *Post hoc* analyses of a large, randomized, placebo-controlled trial focused on smoking cessation revealed that the continuous abstinence rate at 3 months for smokers with diabetes was 24.2%, without any significant adverse reactions noted (Tønnesen et al., 2022; Rojewski et al., 2024). Additionally, no interactions between the GLP-1 agonist dulaglutide and varenicline were observed in a separate study that included diabetic patients (Lengsfeld et al., 2023).

Concerning cytisine, its protonated form is highly hydrophilic becoming more lipophilic in a basic environment, making it soluble in gastrointestinal fluids. However, its gastrointestinal permeability is limited, contributing to low bioavailability. Most pharmacokinetic data derive from *in vivo* studies; in mice, after administration, cytisine takes approximately 2 hours to reach peak blood levels, indicating an absorption rate of 42%. Following oral or intravenous administration, the highest concentrations are found in the liver, bile, adrenal glands, and kidneys. For rabbits, the oral bioavailability stands at 34%, with peak plasma concentrations occurring around 35 min after ingestion. In rats, brain concentrations are lower compared to nicotine or varenicline. This is likely due to cytisine’s low lipophilicity and the possible presence of an unknown brain efflux mechanism. Cytisine does not exceed 30% of its plasma concentration in the brain, whereas nicotine, under similar conditions, reaches 65%. These findings indicate poor penetration of the blood-brain barrier (BBB). Notably, cytisine is excreted unchanged in urine with a half-life (t½) ranging from 37 to 52 min in rabbits. Although human data on cytisine are rather limited, it is still possible to project hypothetical values for some common kinetic parameters. These include a plasma half-life of around 4.8 h on average, a peak plasma concentration of about 1–2 h after dosing, a clearance of between 2 and 5 mL/min/kg, a volume distribution of approximately 1.6 L/kg, a t½ of about 3.6 h, reaching a brain concentration between 2 and 10 nM. In humans, cytisine is excreted unchanged in urine, approximately the 18% 24 h after oral administration, while the percentage reaches about 32% after intravenous administration. A significant limitation to the clinical use of cytisine is its poor ability to cross the BBB, which is considerably lower than that of nicotine or varenicline (Gotti and Clementi, 2021).

Bupropion undergoes metabolism primarily via CYP450, mainly through CYP2B6. CYP1A2, 2A6, 2C9, 2D6, 2E1, and 3A4 isoforms also play a role in the metabolism of bupropion, but to a lesser extent (Connarn et al., 2015). These enzymes are crucial in the metabolic processes of various antidiabetic medications. Therefore, this metabolic overlap may result in altered concentrations and effects of these drugs, necessitating modifications in dosage or the careful selection of antidiabetic treatments for individuals taking bupropion. Additionally, bupropion has been proposed as a potential agent for reducing blood glucose levels, according to evidence from population-based clinical quantitative phenotyping studies (Brown et al., 2018). Furthermore, data from both preclinical and clinical studies have shown that bupropion inhibits the activity of CYP2D6. Therefore, a potential for interaction with antidiabetic drugs metabolized by this isoenzyme exists (Hesse et al., 2000), suggesting that bupropion could have direct or indirect influences on glucose regulation or insulin sensitivity.

Oxidation of bupropion side chain results in the formation of a glycine conjugate of metachlorobenzoic acid, which is then excreted as the major urinary metabolite. Glucuronidated metabolites can also be detected in urine. Although there may be some discrepancies in the literature regarding their different chiral forms, they are formed from all three active metabolites by different UGT enzymes. *In vitro*, the main enzyme responsible for the glucuronidation of hydroxybupropion is UGT2B7, with a minor contribution from UGT2B4. UGT2B7 also plays a predominant role in the formation of erythrohydrobupropion glucuronide, while UGT1A4, UGT1A3, UGT1A9, and UGT2B4 are less involved. Characterization of the UGTs responsible for conjugation of active bupropion metabolites is essential for understanding factors that may influence potential DDIs. *In vitro* experiments have shown that at concentrations up to 200 mcg/ml, 84% of bupropion binds to human plasma proteins. Hydroxybupropion has a similar binding rate, while for threohydrobupropion the extent of protein binding is about half than that of bupropion. Thereby, protein-binding interactions are unlikely to be clinically relevant (Jefferson et al., 2005).

Digital tools highlight that potential DDIs between bupropion and antidiabetics (namely glimepiride, glipizide, glyburide, gliclazide, metformin, sitagliptin, linagliptin, saxagliptin, and canagliflozin) may occur. However, it is important to bear in mind the lack of agreement between the web-based interaction checkers available.

The metabolism of bupropion can be decreased when combined with glimepiride, glyburide, or gliclazide because these drugs are reported to be metabolized by CYP2C9. Thus, co-administration of multiple CYP2C9 substrates can result in competition for the enzyme binding sites, reducing metabolism and increasing plasma levels of one or both affected medications. Elevated plasma levels may result in a higher incidence and/or severity of ADRs (DrugBank, 2024b).

Concerning glipizide, it may decrease the renal excretion rate of bupropion which is likely to lead to higher plasma concentrations. As result, this process may increase the risk, incidence, and/or severity of adverse reactions associated with the augmented exposure to bupropion. Conversely, bupropion may decrease the renal excretion rate of sitagliptin, saxagliptin, and canagliflozin which could result in higher serum concentration. It follows, as before, an increased risk of adverse reactions, in this case related to assumption of these antidiabetics (DrugBank, 2024b).

The renal excretion rate of metformin and linagliptin can be decreased when combined with bupropion. Findings from *in vitro* studies showed that bupropion and its metabolites inhibit OCT2, suggesting that clinically relevant interaction through inhibition of OCT2 may occur at therapeutic bupropion doses. Co-administration of bupropion with OCT2 substrates may result in attenuated OCT2-mediated efflux of those drugs, thereby increasing plasma concentrations (DrugBank, 2024b).

Research on the interaction between bupropion and antidiabetic medications remains limited. In a prospective, placebo-controlled study that examined the impact of bupropion on diabetic men undergoing treatment for hyperglycemia and erectile dysfunction, bupropion administration was found to have no significant effect on diabetes control (Rowland et al., 1997).

A retrospective cohort study demonstrated a reduction in the adherence to oral antidiabetic drugs of approximately 10% or more when administered concomitantly with bupropion (Xing et al., 2018). This percentage represents a significant endpoint, as this reduction in adherence leads to a worse outcome for T2DM patients, resulting in poor glycemic control, diabetes-related hospitalizations, and increased healthcare costs (Balkrishnan et al., 2003; Jha et al., 2012; Oral antidiabetic medication adherence and glycemic control in managed care 2024). Furthermore, the Mayo Clinic database emphasizes the need for caution when using bupropion in patients with T2DM (Bupropion, 2024).

Most studies involving bupropion have focused on obesity and weight loss, particularly in combination with naltrexone. Nevertheless, some insights are applicable. In studies involving overweight and obese patients with type 2 diabetes who were on various oral diabetes medications, the combined therapy of naltrexone and bupropion frequently led to ADRs such as nausea, constipation, vomiting, and diarrhea (Hollander et al., 2013; Apovian, 2016; Chukir et al., 2018; Wharton et al., 2021). Specifically, one study highlighted that patients treated with the naltrexone-bupropion combination who were also taking metformin experienced nausea more frequently (46.2%) compared to those not on metformin (28.2%) (Hollander et al., 2013). However, the rate of serious adverse reactions was low and aligned with rates reported in non-diabetic populations. Possible interactions between antidiabetics and smoking cessation medications are summarized in Table 2.

**TABLE 2:** Possible interactions between antidiabetics and smoking cessation medications, classified according to severity level.

Drug class	Drug	NRT (Ref.)	Bupropion (Ref.)	Varenicline (Ref.)	Cytisine (Ref.)
Sulfonylureas	Glimepiride	Unknown Drug Interaction Checker (2024), INTERCheck Web (2024), DrugBank (2024a)	Minor DrugBank (2024a)	Unknown Drug Interaction Checker (2024), INTERCheck Web (2024), DrugBank (2024a)	Unknown Drug Interaction Checker (2024), INTERCheck Web (2024), DrugBank (2024a)
	Glipizide	Unknown Drug Interaction Checker (2024), INTERCheck Web (2024), DrugBank (2024a)	Minor DrugBank (2024a)	Minor DrugBank (2024a)	Unknown Drug Interaction Checker (2024), INTERCheck Web (2024), DrugBank (2024a)
	Glyburide	Unknown Drug Interaction Checker (2024), INTERCheck Web (2024), DrugBank (2024a)	Moderate DrugBank (2024a)	Unknown Drug Interaction Checker (2024), INTERCheck Web (2024), DrugBank (2024a)	Unknown Drug Interaction Checker (2024), INTERCheck Web (2024), DrugBank (2024a)
	Gliclazide	Unknown Drug Interaction Checker (2024), INTERCheck Web (2024), DrugBank (2024a)	Minor DrugBank (2024a)	Unknown Drug Interaction Checker (2024), INTERCheck Web (2024), DrugBank (2024a)	Unknown Drug Interaction Checker (2024), INTERCheck Web (2024), DrugBank (2024a)
Glinides	Mitiglinide	Unknown Drug Interaction Checker (2024), INTERCheck Web (2024), DrugBank (2024a)	Unknown Drug Interaction Checker (2024), INTERCheck Web (2024), DrugBank (2024a)	Unknown Drug Interaction Checker (2024), INTERCheck Web (2024), DrugBank (2024a)	Unknown Drug Interaction Checker (2024), INTERCheck Web (2024), DrugBank (2024a)
	Repaglinide	Unknown Drug Interaction Checker (2024), INTERCheck Web (2024), DrugBank (2024a)	Unknown Drug Interaction Checker (2024), INTERCheck Web (2024), DrugBank (2024a)	Unknown Drug Interaction Checker (2024), INTERCheck Web (2024), DrugBank (2024a)	Unknown Drug Interaction Checker (2024), INTERCheck Web (2024), DrugBank (2024a))
Biguanides	Metformin	Moderate DrugBank (2024a)	Moderate DrugBank (2024a)	Moderate DrugBank (2024a)	Unknown Drug Interaction Checker (2024), INTERCheck Web (2024), DrugBank (2024a)
Thiazolidindions	Pioglitazone	Unknown Drug Interaction Checker (2024), INTERCheck Web (2024), DrugBank (2024a)	Unknown Drug Interaction Checker (2024), INTERCheck Web (2024), DrugBank (2024a)	Unknown Drug Interaction Checker (2024), INTERCheck Web (2024), DrugBank (2024a)	Unknown Drug Interaction Checker (2024), INTERCheck Web (2024), DrugBank (2024a)
α-glucosidase inhibitors	Acarbose	Unknown Drug Interaction Checker (2024), INTERCheck Web (2024), DrugBank (2024a)	Unknown Drug Interaction Checker (2024), INTERCheck Web (2024), DrugBank (2024a)	Unknown Drug Interaction Checker (2024), INTERCheck Web (2024), DrugBank (2024a)	Unknown Drug Interaction Checker (2024), INTERCheck Web (2024), DrugBank (2024a)
GLP-1 Receptor Agonist	Exenatide	Unknown Drug Interaction Checker (2024), INTERCheck Web (2024), DrugBank (2024a)	Unknown Drug Interaction Checker (2024), INTERCheck Web (2024), DrugBank (2024a)	Unknown Drug Interaction Checker (2024), INTERCheck Web (2024), DrugBank (2024a)	Unknown Drug Interaction Checker (2024), INTERCheck Web (2024), DrugBank (2024a)
	Liraglutide	Unknown Drug Interaction Checker (2024), INTERCheck Web (2024), DrugBank (2024a)	Unknown Drug Interaction Checker (2024), INTERCheck Web (2024), DrugBank (2024a)	Unknown Drug Interaction Checker (2024), INTERCheck Web (2024), DrugBank (2024a)	Unknown Drug Interaction Checker (2024), INTERCheck Web (2024), DrugBank (2024a)
	Dulaglutide	Unknown Drug Interaction Checker (2024), INTERCheck Web (2024), DrugBank (2024a)	Unknown Drug Interaction Checker (2024), INTERCheck Web (2024), DrugBank (2024a)	Unknown Drug Interaction Checker (2024), INTERCheck Web (2024), DrugBank (2024a)	Unknown Drug Interaction Checker (2024), INTERCheck Web (2024), DrugBank (2024a)
	Semaglutide	Unknown Drug Interaction Checker (2024), INTERCheck Web (2024), DrugBank (2024a)	Unknown Drug Interaction Checker (2024), INTERCheck Web (2024), DrugBank (2024a)	Unknown Drug Interaction Checker (2024), INTERCheck Web (2024), DrugBank (2024a)	Unknown Drug Interaction Checker (2024), INTERCheck Web (2024), DrugBank (2024a)
DPP-4 Inhibitors	Sitagliptin	Unknown Drug Interaction Checker (2024), INTERCheck Web (2024), DrugBank (2024a)	Minor DrugBank (2024a)	Minor DrugBank (2024a)	Unknown Drug Interaction Checker (2024), INTERCheck Web (2024), DrugBank (2024a)
	Linagliptin	Moderate DrugBank (2024a)	Moderate DrugBank (2024a)	Minor DrugBank (2024a)	Unknown Drug Interaction Checker (2024), INTERCheck Web (2024), DrugBank (2024a)
	Vildagliptin	Unknown Drug Interaction Checker (2024), INTERCheck Web (2024), DrugBank (2024a)	Unknown Drug Interaction Checker (2024), INTERCheck Web (2024), DrugBank (2024a)	Unknown Drug Interaction Checker (2024), INTERCheck Web (2024), DrugBank (2024a)	Unknown Drug Interaction Checker (2024), INTERCheck Web (2024), DrugBank (2024a)
	Saxagliptin	Unknown Drug Interaction Checker (2024), INTERCheck Web (2024), DrugBank (2024a)	Minor DrugBank (2024a)	Minor DrugBank (2024a)	Unknown Drug Interaction Checker (2024), INTERCheck Web (2024), DrugBank (2024a)
SGLT-2 inhibitors	Canagliflozin	Unknown Drug Interaction Checker (2024), INTERCheck Web (2024), DrugBank (2024a)	Minor DrugBank (2024a)	Minor DrugBank (2024a)	Unknown Drug Interaction Checker (2024), INTERCheck Web (2024), DrugBank (2024a)
	Empagliflozin	Unknown Drug Interaction Checker (2024), INTERCheck Web (2024), DrugBank (2024a)	Unknown Drug Interaction Checker (2024), INTERCheck Web (2024), DrugBank (2024a)	Unknown Drug Interaction Checker (2024), INTERCheck Web (2024), DrugBank (2024a)	Unknown Drug Interaction Checker (2024), INTERCheck Web (2024), DrugBank (2024a)
	Dapagliflozin	Unknown Drug Interaction Checker (2024), INTERCheck Web (2024), DrugBank (2024a)	Unknown Drug Interaction Checker (2024), INTERCheck Web (2024), DrugBank (2024a)	Unknown Drug Interaction Checker (2024), INTERCheck Web (2024), DrugBank (2024a)	Unknown Drug Interaction Checker (2024), INTERCheck Web (2024), DrugBank (2024a)

Major highly clinically significant. Avoid combinations; the risk of the interaction outweighs the benefit. Moderate: moderately clinically significant. Usually avoid combinations; use it only under special circumstances. Minor: minimally clinically significant. Minimize risk; assess risk and consider an alternative drug, take steps to circumvent the interaction risk and/or institute a monitoring plan. Unknown: no interaction information available.

## 6 Discussion

The intricacies of polypharmacy in managing patients with multiple chronic conditions pose a significant challenge. Chronic diseases like diabetes mellitus require detailed management to track disease progression and monitor potential interactions between medications and external factors, such as cigarette smoking. Indeed, smoking and diabetes are among the most prevalent health issues, collectively impacting the general population. Given the overlap between diabetes and smoking habits, it is crucial for patients’ health to consider potential interactions between smoking and smoking cessation and the metabolism of antidiabetic drugs.

Tobacco smoke, through the induction of CYP450 enzymes and UGTs, may modulate the pharmacokinetics of antidiabetic medications. Notably, compounds such as PAHs within cigarette smoke emerge as principal culprits, expediting the metabolism of drugs such as metformin, linagliptin, and glyburide, which can consequently impair their therapeutic efficacy. Furthermore, this review highlights the lack of literature addressing the comprehensive effects of smoking and smoking cessation in diabetic patients. Despite smoking’s extensive impact on global health, particularly exacerbating conditions such as diabetes, the intersections between cigarette smoking, cessation efforts, and diabetes management remain underexplored. This gap underscores a critical need for further research to refine our understanding with a focus on treatment protocols designed for diabetic patients who smoke.

This review underlines the intricate DDIs between smoking cessation aids and antidiabetic medications, underpinning their significant impact on treatment outcomes for diabetic patients trying to quit smoking. It stresses the importance of personalized treatment plans, rooted in a deep understanding of these interactions, to facilitate successful smoking cessation and effective diabetes management. Healthcare professionals are urged to be vigilant about potential changes in drug efficacy among diabetic smokers undergoing cessation. They may consider these interactions when prescribing and managing antidiabetic treatments, making necessary dosage adjustments to prevent suboptimal drug concentrations and maintain therapeutic effectiveness.

In conclusion, this scoping review sheds light on the critical intersections of smoking, smoking cessation, and antidiabetic drug metabolism, and calls for an augmented focus on research, clinical awareness, and patient-centered interventions. Such approaches are essential for optimizing diabetes management strategies and improving the overall health outcomes for this vulnerable population segment.

## DiaSmokeFree Working Group

Agostino Di Ciaula, Clinica Medica “A. Murri”, Department of Precision and Regenerative Medicine and Ionian Area (DiMePre-J), University “Aldo Moro” Medical School, Bari–Italy. Tabinda Dugal, Royal Cornwall Hospital NHS Trust consultant endocrinologist and lead for diabetes renal, diabetes retinal, transition endocrine service, Treliske, Truro, United Kingdom, International faculty department of Endocrinology College of Physicians and Surgeons. Andre Kengne, Non-Communicable Diseases Research Unit, South African Medical Research Council and University of Cape Town, Cape Town, South Africa, Department of Biological and Environmental Science, Faculty of Science, Walter Sisulu University, Mthatha, South Africa. Phuong Le Dinh, General Practice, Family Medicine and Check-up Department, FV Hospital Ho Chi Minh City, Vietnam. Anoop Misra, Diabetes Foundation (India), New Delhi, India, National Diabetes, Obesity and Cholesterol Foundation (N-DOC), New Delhi, India, Fortis C-DOC Centre for Excellence for Diabetes, Metabolic Disease, and Endocrinology, New Delhi, India. Riccardo Polosa, Center of Excellence for the Acceleration of HArm Reduction (CoEHAR), University of Catania, Italy, Centre for the Prevention and Treatment of Tobacco Addiction (CPCT), Catania, Italy, Department of Clinical & Experimental Medicine, University of Catania, Catania, Italy. Syed Abbas Raza, Consultant Endocrinologist, Shaukat Khanum Cancer Hospital and Research Center, Peshawar. Cristina Russo, Ashford and Saint Peter’s Hospitals NHS Foundation Trust, Chertsey KT16 0PZ, United Kingdom. Roberta Sammut, Senior Lecturer, Faculty of Health Sciences, University of Malta, Malta, Associate Editor, International Journal of Nursing Studies, Member of the Management Board of id-Dar tal-Providenza, Siggiewi, Malta. Noel Somasundaram, Consultant Endocrinologist Diabetes and Hormone Center, Colombo. President, South Asian Federation of Endocrine Societies. Chief Endocrinologist, National Hospital of Sri Lanka. President, Endocrine Society of Sri Lanka. Magda Walicka, Department of Human Epigenetics, Mossakowski Medical Research Institute, Polish Academy of Sciences, 5 Pawi&nacute;skiego str, 02-106 Warsaw, Poland, Department of Internal Diseases, Endocrinology and Diabetology, The National Institute of Medicine of the Ministry of Interior and Administration, 137 Wołoska str., 02-507 Warsaw, Poland.

## Abbreviations

ADA, American Diabetes Association; ADRs, Adverse Drug Reactions; AUC, Area Under the Curve; BBB: Blood-Brain Barrier; CNS, Central Nervous System; CO, Carbon Monoxide; COPD, Chronic Obstructive Pulmonary Disease; CYP450, Cytochrome P450; DDIs, Drug-Drug Interactions; DPP-4, Dipeptidyl Peptidase-4; EMA, European Medicines Agency; FDA, Food and Drug Administration; GBD, Global Burden of Disease; GLP-1, Glucagon-Like Peptide-1; HEK-293, Human Embryonic Kidney Cell Line; NRT, Nicotine-Replacement Therapy; OATP, Organic Anion Transporting Polypeptide; OCT, Organic Cation Transporter; PAHs, Polycyclic Aromatic Hydrocarbons; SGLT-2, Sodium-Glucose Linked Transporter-2; SmPC, Summary of Product Characteristic; SUs, Sulfonylureas; T2DM, Type 2 Diabetes Mellitus; UDP, Uridine Diphoshate; UGTs, UDP-GlucuronosylTransferase.
