# Supplementary material for: A web-based scoping review assessing the influence of smoking and smoking cessation on antidiabetic drug meabolism: implications for medication efficacy
Source: Front Pharmacol. 2024 Jun 18;15:1406860. doi: 10.3389/fphar.2024.1406860 (PMC11217182; doi:10.3389/fphar.2024.1406860)
Supplement: Supplementary file 1 [file Table1.DOCX]

Supplementary Material

# Supplementary Table

**Table**: Search string details adopted for each database.

| **Database** | |
| --- | --- |
| **PubMed** | **Embase** |
| (diabetes mellitus, type 2[MeSH Terms]) OR (type 2 diabetes mellitus[Title/Abstract]) OR (T2DM[Title/Abstract]) OR (type II diabetes mellitus[Title/Abstract]) OR (type 2 diabetes[Title/Abstract]) OR (diabetes[Title/Abstract]) OR (diabetic[Title/Abstract]) AND (smoking[MeSH Terms]) OR (smoking[Title/Abstract]) OR (smoke[Title/Abstract]) OR (cigarette[Title/Abstract]) OR (tobacco[MeSH Terms]) OR (tobacco products[MeSH Terms]) OR (tobacco[Title/Abstract]) AND (antidiabetic drugs[Title/Abstract]) OR (hypoglycemic agents[MeSH Terms]) OR (hypoglycemic agents[Title/Abstract]) OR (insulin[MeSH Terms]) OR (insulin[Title/Abstract]) OR (glimepiride[Title/Abstract]) OR (glimepiride[MeSH Terms]) OR (glipizide[Title/Abstract]) OR (glipizide[MeSH Terms]) OR (glyburide[Title/Abstract]) OR (glyburide[MeSH Terms]) OR (gliclazide[Title/Abstract]) OR (gliclazide[MeSH Terms]) OR (mitiglinide[Title/Abstract]) OR (mitiglinide[MeSH Terms]) OR (repaglinide[Title/Abstract]) OR (repaglinide[MeSH Terms]) OR (metformin[Title/Abstract]) OR (metformin[MeSH Terms]) OR (pioglitazone[Title/Abstract]) OR (pioglitazone[MeSH Terms]) OR (acarbose[Title/Abstract]) OR (acarbose[MeSH Terms]) OR (exenatide[Title/Abstract]) OR (exenatide[MeSH Terms]) OR (liraglutide[Title/Abstract]) OR (liraglutide[MeSH Terms]) OR (dulaglutide[Title/Abstract]) OR (dulaglutide[MeSH Terms]) OR (semaglutide[Title/Abstract]) OR (semaglutide[MeSH Terms]) OR (sitagliptin[Title/Abstract]) OR (sitagliptin[MeSH Terms]) OR (linagliptin[Title/Abstract]) OR (linagliptin[MeSH Terms]) OR (vildagliptin[Title/Abstract]) OR (vildagliptin[MeSH Terms]) OR (saxagliptin[Title/Abstract]) OR (saxagliptin[MeSH Terms]) OR (canagliflozin[Title/Abstract]) OR (canagliflozin[MeSH Terms]) OR (empagliflozin[Title/Abstract]) OR (empagliflozin[MeSH Terms]) OR (dapagliflozin[Title/Abstract]) OR (dapagliflozin[MeSH Terms]) AND (nicotine replacement therapies[Title/Abstract]) OR (nicotine replacement therapies[MeSH Terms]) OR (nicotine replacement products[Title/Abstract]) OR (nicotine replacement products[MeSH Terms])) OR (NRT[Title/Abstract])) OR (NRT[MeSH Terms]) OR (NRTs[Title/Abstract]) OR (NRTs[MeSH Terms]) OR (varenicline[Title/Abstract]) OR (varenicline[MeSH Terms]) OR (cytisine[Title/Abstract]) OR (cytisine[MeSH Terms]) OR (bupropion[Title/Abstract]) OR (bupropion[MeSH Terms]) AND (cytochrome P450[Title/Abstract]) OR (cytochrome P450[MeSH Terms]) OR (cytochrome p450 enzyme system[Title/Abstract]) OR (cytochrome p450 enzyme system[MeSH Terms]) OR (CYP[Title/Abstract]) OR (CYP[MeSH Terms]) OR (uridine5'-diphospho-glucuronosyltransferase[Title/Abstract]) OR (uridine5'-diphospho-glucuronosyltransferase[MeSH Terms]) OR (UGT[Title/Abstract]) OR (UGT[MeSH Terms]) | #1 'Type 2 Diabetes '/exp OR 'Type 2 Diabetes Mellitus'/exp #2 'smoke'/exp OR 'smoking'/exp OR 'smokers'/exp OR 'tobacco'/exp OR 'cigarette'/exp  #3 'anti-diabetic drugs'/exp OR 'hypoglycemic agents'/exp OR 'insulin'/exp OR 'glimepiride'/exp OR 'glipizide'/exp OR 'glyburide'/exp OR 'gliclazide'/exp OR 'mitiglinide'/exp OR 'repaglinide'/exp OR 'Metformin'/exp OR 'Pioglitazone'/exp OR 'Acarbose'/exp OR 'Exenatide'/exp OR 'Liraglutide'/exp OR 'Dulaglutide'/exp OR 'Semaglutide'/exp OR 'Sitagliptin phosphate'/exp OR 'Linagliptin'/exp OR 'Vildagliptin'/exp OR 'Saxagliptin'/exp OR 'Canagliflozin'/exp OR 'Empagliflozin'/exp OR 'Dapagliflozin'/exp  #4'Cytochrome P450'/exp OR 'CYP'/exp OR 'Uridine 5'-diphospho-glucuronosyltransferase'/exp OR 'UGT'/exp  #1 AND #2 AND #3 AND #4 |
